# Host Defense Peptides as Templates for Antifungal Drug Development

**DOI:** 10.3390/jof6040241

**Published:** 2020-10-23

**Authors:** Virginia Basso, Dat Q. Tran, André J. Ouellette, Michael E. Selsted

**Affiliations:** 1 Department of Pathology and Laboratory Medicine, Keck School of Medicine, University of Southern California, Los Angeles, CA 90089, USA; Virginia.Basso@med.usc.edu (V.B.); dtran@oryntherapeutics.com (D.Q.T.); aouellet@med.usc.edu (A.J.O.); 2 Oryn Therapeutics, Vacaville, CA 95688, USA; 3 Norris Comprehensive Cancer Center of the University of Southern California, Los Angeles, CA 90089, USA

**Keywords:** antifungals, host defense peptides, peptidomimetics, immunomodulation, multidrug resistance, defensins

## Abstract

Current treatment for invasive fungal diseases is limited to three classes of antifungal drugs: azoles, polyenes, and echinocandins. The most recently introduced antifungal class, the echinocandins, was first approved nearly 30 years ago. The limited antifungal drug portfolio is rapidly losing its clinical utility due to the inexorable rise in the incidence of invasive fungal infections and the emergence of multidrug resistant (MDR) fungal pathogens. New antifungal therapeutic agents and novel approaches are desperately needed. Here, we detail attempts to exploit the antifungal and immunoregulatory properties of host defense peptides (HDPs) in the design and evaluation of new antifungal therapeutics and discuss historical limitations and recent advances in this quest.

## 1. Introduction

Invasive fungal infections are a global health concern, with high rates of incidence and mortality, particularly in immunocompromised patients [1,2,3]. The paucity of antifungal drugs available for treatment of invasive mycoses represents a growing public health vulnerability, as the numbers of immunosuppressed individuals grow with increasing use of cancer chemotherapy, bone marrow and solid organ transplants, and immunosuppressive drugs for treatment of autoimmune disorders. Serious, often life-threatening, mycoses that affect humans are primarily due to only a few genera (Table 1). *Candida* spp. infections range from mild and superficial to invasive infections with mortality rates of 40% or greater [2]. An estimated 200,000 cases of *Aspergillus* spp. infections occur annually with invasive disease primarily affecting immunocompromised patients. With treatment, invasive aspergillosis causes death in up to 50% of affected patients, whereas left untreated, as often occurs in developing countries, lethality approaches 100% [1]. Immunocompromised patients are also highly susceptible to cryptococcosis caused by *Cryptococcus neoformans* and closely related *Cryptococcus gattii*. Although cryptococcosis is initially a pulmonary infection, in the immunosuppressed patient the organism commonly disseminates systemically to the central nervous system (CNS) causing meningoencephalitis with an estimated 70% mortality [1].

Currently, there are three classes of antifungals: azoles, polyenes, and echinocandins (Table 2). Azoles are the most widely used class of antifungal agents because of their bioavailability as oral and intravenous (i.v.) formulations and their excellent safety profiles. However, some *Candida* species, such as *C. krusei*, are intrinsically resistant to fluconazole, the most widely used azole. Fluconazole-resistant isolates are emerging rapidly because azoles are fungistatic which promotes resistance selection with extended use [4,5]. Polyenes have been in use since the 1950s, but their toxicities limit their use to severe infections. The toxicity of amphotericin B (AmpB) is well known, particularly its nephrotoxicity, due to its affinity for and disruption of host cell membranes [6]. Fortunately, lipid-associated and liposomal formulations of AmpB have reduced adverse events associated with its use [6]. Although initially approved nearly 30 years ago, echinocandins are the newest class of antifungals. While usually effective in treatment of candidiasis and aspergillosis [7,8], echinocandins are ineffective against *Cryptococcus* spp. and endemic mycoses [7].

The public health impact of systemic mycoses is amplified by the increasing incidence of multidrug resistant (MDR) pathogens. Numerous clinical isolates have emerged that are resistant to all three classes of approved antifungals [4,9,10,11,12,13], highlighted by the international spread of MDR *C. auris,* which is now classified by CDC as a serious threat [14,15]. In the United States alone, systemic mycoses are estimated to cause more than 1.6 million deaths annually [16] with associated treatment costs exceeding USD 7 billion [17]. Accordingly, new efforts are underway to address the growing and worldwide impact of systemic fungal infections, including the development of fungal vaccines and new therapeutic strategies [18,19,20]. Here we discuss the potential of host defense peptides (HDPs) (also long referred to as antimicrobial peptides) as bioinspired templates for new agents to treat invasive fungal infections. While antimicrobial peptides are expressed by nearly all life forms, the primary focus of this review will be on antifungal HDPs expressed as elements of the mammalian immune system.

## 2. Mammalian HDPs

HDPs are short polypeptides, generally fewer than 50 amino acids, that display a wide range of in vitro antimicrobial activities against bacteria, fungi, viruses, and unicellular protozoa [21]. HDPs expressed in mammals are structurally diverse, predominantly expressed in leukocytes and epithelia [22,23,24], and nearly all were identified by in vitro antimicrobial screens of fractionated tissue extracts. A number of elegant studies have disclosed a central role for HDPs in innate immunity [25,26,27,28,29], thus it was logical that these molecules be evaluated for their potential as therapeutics. In addition, enzymatic cleavage of larger proteins may generate peptides (termed cryptides) with antimicrobial properties that potentially contribute to antimicrobial host defense [30]. Collectively, studies that began in the 1960s have identified several hundred antimicrobial peptides, many of which possess antifungal activities [18,31]. As discussed below, the vast majority of mammalian antifungal HDPs are fungicidal. Moreover, many HDPs also have immunoregulatory properties that appear to be essential for antifungal responses of host cells in vitro and for antifungal efficacy in vivo. In the following sections, we detail the major classes of mammalian antifungal HDPs, the status of their development as therapeutic candidates, and discuss promising advances of HDP-inspired, antifungal peptidomimetics.

### 2.1. Histatins

Histatins (Hsts; Table 3) constitute a family of linear histidine-rich cationic peptides isolated from human saliva [32]. Among the 12 Hst family members characterized, Hst5 is the most potent fungicidal peptide against *C. albicans* in vitro [33]. Synthetic Hst5 variants, termed Dhar4 and Dhar5, also inhibit biofilm formation of MDR *C. albicans* in vitro [34,35]. The candidacidal activity of Hst5 results from its binding to the yeast cell surface followed by internalization into the cytosol. Intracellular Hst5 induces oxidative stress and impairs mitochondrial function that induces a lethal ionic imbalance in fungal cells [33]. PAC-113 is a dodecapeptide, corresponding to amino acids 4–15 of Hst5, in which the C-terminal His is carboxamidated. Topically applied PAC-113 has been shown to be effective in phase I and II in clinical trials for oral candidiasis in HIV patients [18], and a rinse formulation for treatment and prevention of oral candidiasis is being developed by Pacgen Biopharmaceuticals. PAC-113 is currently marketed as an over-the-counter product in Taiwan. As topical agents, Hst and derivatives thereof have shown promise as candidacidal agents with low toxicity toward mammalian cells [36]. There have been no reports of systemic delivery of Hsts or their derivatives, indicating their utility is limited to topical applications.

### 2.2. Cathelicidins

The cathelicidin peptide family is a heterogeneous group of peptides with sizes ranging from 12 to 80 residues with highly diverse sequences and structures [73]. They share a highly conserved, amino terminal prepro sequence termed the cathelin domain. The most extensively studied antifungal cathelicidin is LL-37 (Table 3), the only known human cathelicidin. LL-37 is a cationic α-helical peptide composed of 37 amino acids that is widely expressed in the skin, gastrointestinal, urinary, and respiratory tracts [74,75], as well as by monocytes, neutrophils, NK, B, and T cells [76,77,78]. LL-37 is fungicidal in vitro against *C. albicans* and *Aspergillus* spp. [39,79]. LL-37 disrupts the cytoplasmic membrane of *C. albicans* blastospores [39] and also protects mammalian host cells by inhibiting biofilm formation and host cell binding by interaction with yeast cell wall carbohydrates [80]. LL-37 inhibits *A. fumigatus* hyphal growth and adhesion to host cells resulting in suppression of TNF-α and IL-6 release [42,45]. The immunoregulatory effects of LL-37 include its chemoattraction of neutrophils [38] and augmentation of reactive oxygen species (ROS) production by neutrophils exposed to phorbol 12-myristate 13-acetate or bacteria [37]. The peptide also induces chemokine release by leukocytes and epithelial cells, in particular IL-8, to increase neutrophil recruitment [40], and it inhibits neutrophil apoptosis [43]. LL-37 selectively modulates proinflammatory cytokines by peripheral blood mononuclear cells (PBMCs). For example, the peptide downregulates TNFα, IL-1β, IL-6, and IL-8 secretion by blood monocytes stimulated with TLR2 or TLR4 agonists, but it stimulates TNFα and IL-6 production by CpG-stimulated monocytes [20,41,44].

Other cathelicidins evaluated for their antifungal potential include porcine protegrins (cationic hairpin peptides of 16–18 amino acids stabilized by two disulfides) [46] and bovine indolicidin, a cationic tryptophan-rich tridecapeptide amide [81] (Table 3). Protegrins and indolicidin are expressed at high levels in granulocytes of the respective species. Protegrins have potent fungicidal activity against *C. albicans* in vitro but failed to advance clinically due to their unacceptable toxicity toward mammalian cells [46]. Similarly, indolicidin was fungicidal against *Candida* spp. and *C. neoformans* in vitro [82], but was cytotoxic and poorly tolerated in naive BALB/c mice that received the peptide i.v. [47]. However, liposomal encapsulation of indolicidin markedly reduced cytotoxicity and greatly improved tolerability when administered i.v. to mice [47]. This improved formulation was tested for therapeutic efficacy in mice challenged with *A. fumigatus* spores. A single dose of liposomal indolicidin was therapeutically effective in this model, providing evidence that liposomal formulation technology, used to enhance tolerability and efficacy of AmpB, could be applied to an antifungal peptide [47].

### 2.3. α- and β-Defensins

The defensin family comprises three structural subfamilies, termed α-, β-, and θ-defensins [83], the first two of which will be discussed here. α- and β-defensins vary in length from 29 to 47 amino acids [84], and despite having different tri-disulfide motifs, have remarkably similar tertiary structures (Table 3). In vitro, neutrophil α-defensins are fungicidal against numerous species, including *C. albicans* [85], *C. neoformans* [86], *Coccidioides immitis* [87], *Rhizopus oryzae*, and *A. fumigatus* [88]. Additionally, numerous studies demonstrate that human α-defensins possess immunomodulatory properties that are predominantly proinflammatory. For example, α-defensins induce IL-6 secretion by T-cells [50], upregulate TNFα and IL-1β expression by monocytes [48], stimulate TNFα, IL-6, and IL-12 expression in myeloid dendritic cells [89], and induce IL-8 release by lung epithelial cells [49,51,90]. α-defensins also act as a bridge between innate and acquired immunity by promoting antigen or tumor-specific immune responses in vitro and in vivo [50,91,92], mobilizing immature dendritic cells and naïve T lymphocytes [93], and enhancing chemotaxis of macrophages, mast cells, and T lymphocytes [94].

β-defensins were first identified in cattle [95,96], and soon thereafter discovered in humans [97]. There are more than 40 human β-defensin (HBD) genes [98], but most studies on HBD peptides have focused on HBDs 1–3 which are expressed in many tissues [99]. HBD-2 and 3 expression is induced by proinflammatory signals, such as TNFα and IL-1β [100,101,102,103,104,105], whereas HBD-1 is expressed constitutively [106,107]. HBD-1 was fungistatic against *Candida* spp. [108,109] whereas both HBD-2 and HBD-3 were fungicidal against *C. albicans* [55]. In addition to their microbicidal properties, like α-defensins, β-defensins function as immunostimulants. For example, mouse β-defensin-2 is an endogenous ligand for TLR-4 on immature dendritic cells, producing a Th1 polarized immune response with production of IL-1 α/β, IL-6, and IL-12 [52]. HBDs 2–4 induced expression of several proinflammatory cytokines in keratinocytes [53,54] and HBD-2 elicited similar effects in CD3/28-stimulated peripheral T-cells [53]. Under some conditions, HBDs are reported to have immunosuppressive activities [110].

Therapeutic development of α- and β-defensins, including those expressed in humans, have not advanced to the clinic for any indication. In fact, none of the antifungal peptides described above have been translated clinically for treatment of invasive mycoses. However, Hst and indolicidin analogs, LL-37-derived peptides, and a number of other synthetic peptides are under development as topical agents to treat a number infectious diseases including mucocutaneous candidiasis [31]. As discussed by Haney et al., it is not surprising that the antimicrobial activities of HDPs determined in vitro do not predictably translate functionally in the physiologic milieu of infected host tissues [111], despite the wide use of this approach for small molecules. For HDPs, the challenge of in vitro-to-in vivo translation, especially for a systemically administered drug, is complicated by other factors including unpredictable binding of the peptide to blood elements and tissues, peptide degradation, unfavorable pharmacokinetics, and potentially adverse effects of the HDP itself. It is evident, therefore, that preclinical efficacy and toxicology studies are required to justify clinical development of HDPs.

## 3. Potential Role of HDPs in Antifungal Drug Development

For reasons outlined above, the track record of peptide-based drug development of anti-infectives is littered with failures. Ironically, the development of peptide drugs in general is on a steep positive trajectory, with 20 new peptide-based clinical trials every year, and more than 400 peptidic drugs in clinical development. Over the last 20 years, a total of 60 peptide drugs have been approved worldwide, with metabolic disorders and cancer as the main targeted therapeutic areas [112]. Moreover, as noted above, a number of HDPs are in development for topical antifungal applications, including the treatment of oral and vulvovaginal candidiasis [31]. Despite the challenges of antifungal drug development in general, and the inherent barriers encountered in developing peptide drugs, there is reason to be hopeful that the biological properties of HDPs may contribute to the quest for new antifungal agents. The following sections provide a few examples of recent successes, exploiting both the antifungal and immunotherapeutic properties of HDPs.

### 3.1. IDR-1018

Hancock and colleagues were among the first to recognize that the antimicrobial efficacy of HDPs was linked to the immunoregulatory properties of the molecule [111,113]. This informed the synthesis of innate defense regulator (IDR) peptides using bovine cathelicidin Bac2a as the template for analog design and subsequent activity screens. Freitas et al. demonstrated the utility of this approach in studies of IDR-1018, a linear dodecapeptide amide that was tested for its effect on survival in a mouse model of systemic candidiasis [56]. Candidemic mice were treated daily with 1, 5, or 10 mg/kg of IDR-1018 by the i.p. route beginning 24 h post retroorbital infection. The highest dose statistically prolonged survival and had a modest effect on fungal kidney burden after 7 days of peptide treatment. It was concluded that the therapeutic effect of IDR-1018 is mediated by its modulation of proinflammatory cytokines (TNFα, IL-1β, IL-6, and IL-12) by bone marrow derived macrophages stimulated with heat killed *C. albicans* [56].

### 3.2. hLF 1-11

Human lactoferrin (hLF) is a 77 kDa iron-binding glycoprotein implicated in host defense by direct killing of *C. albicans*, or through the action of proteolytic fragments (cryptides). A number of studies were performed using hLF1-11, the amino terminal undecapeptide of hLF. hLF1-11 is fungicidal in vitro, having greater potency than hLF itself [59]. Direct fungal killing occurred by increasing mitochondrial potential and permeability via calcium uptake, which resulted in ROS and adenosine triphosphate production, and consequently in *C. albicans* cell death [58]. The efficacy of hLF1-11 in vivo was evaluated in neutropenic mice challenged i.v. with *C. albicans* Y01-19 and treated with a single i.v. dose of hLF1-11 24 h post infection. After an additional 18 h, mice were euthanized and renal fungal burden was quantified as a function of peptide dose. Significant reduction is fungal burden was obtained with peptide doses as low as 0.4 ng/kg [58]. Although the study design did not allow for determination of treatment impact on survival, histologic analysis indicated that peptide treatment inhibited filamentation of surviving fungi. Moreover, hLF1-11 treatment dose-dependently reduced serum TNFα and IL-6 in infected mice.

### 3.3. θ-Defensins

Recent studies suggest that θ-defensins, the third of the three mammalian defensin subfamilies, may represent a unique HDP template for antifungal drug development. θ-Defensins are cyclic octadecapeptides expressed exclusively in Old World monkeys and are the only known cyclic polypeptides in the Animal Kingdom [114]. The cyclic backbone is produced by head-to-tail splicing of two nonapeptides derived from α-defensin-like precursors [115], and binary combinations produce multiple θ-defensin isoforms (e.g., six in rhesus macaques [116]; 10 in olive baboons [117]) that have distinct antimicrobial activities against bacteria and fungi in vitro [9,116,117,118].

Rhesus theta defensin 1 (RTD-1), the first θ-defensin isoform described, has served as the prototype for characterizing in vitro antimicrobial activities against bacteria, fungi, and enveloped viruses [9,85,117,118,119,120,121,122]. In early in vivo studies, single dose administration of RTD-1 was effective in *E. coli*- and polymicrobial sepsis in BALB/c mice [62]. While RTD-1 is broadly microbicidal in vitro, numerous lines of evidence suggested that in vivo efficacy is a function of RTD-1 immunomodulation of systemic inflammation. Studies show that RTD-1 homeostatically regulates LPS- and *E. coli*-stimulated release of TNFα by inhibiting tumor necrosis alpha converting enzyme (TACE; ADAM17) [61], as well as other proinflammatory cytokines by regulation of nuclear factor kappa B and mitogen-activated protein kinase pathways [64]. These studies suggested that RTD-1 promotes survival in mouse models of sepsis by immunomodulation of the host response.

In a recent study, the candidacidal properties and fungicidal mechanisms of natural θ-defensin isoforms were evaluated, demonstrating that the peptides are broadly, though differentially, fungicidal against numerous *Candida* species including MDR resistant strains of *C. albicans* and *C. auris* [9]. RTD-1 killing of *C. albicans* was time and concentration dependent, and temporally correlated with yeast permeabilization, ATP release, and induction of fungal ROS [9]. RTD-1 was >200-fold more active than Hst5 in these studies, and was stable to fungal proteases that rapidly degraded Hst5 [9]. These findings are consistent with previous studies demonstrating the stability of RTD-1 in biological matrices [62]. Moreover, θ-defensins are extremely poor immunogens [63], suggesting that they, or analogs thereof, might have utility as systemically administered therapeutics.

Since RTD-1 was among the most potent and broadly anti-candidal θ-defensins in vitro [9], this peptide was evaluated for therapeutic efficacy in a mouse model of systemic candidiasis. Mice were challenged i.v. with drug sensitive or MDR *C. albicans*, and treated i.p. with 5 mg/kg of RTD-1 once daily for 7 days beginning 24 post infection [60]. RTD-1 was highly effective in promoting long term survival of mice infected with both *C. albicans* strains, and this was statistically equivalent to the effects of fluconazole and caspofungin in mice infected with drug sensitive *C. albicans* SC5314 [60]. In mice infected with MDR *C. albicans* 53264, endpoint analysis showed RTD-1 to be superior to both fluconazole and caspofungin [60]. In both infections, RTD-1 was highly effective in promoting fungal clearance, as determined by quantitation of renal fungal burden, and was more effective in promoting fungal clearance than fluconazole or caspofungin [60]. Moreover, RTD-1 treatment homeostatically restored dysregulated inflammatory responses, including chronically elevated granulocyte counts and inflammatory cytokines responses (TNFα, IL-1β, IL-6, IL-10, and IL-17) that occurred in sham-treated infected mice [60]. Interestingly, RTD-1 treatment produced a transient 3–4-fold increase in peripheral blood neutrophils in infected animals [60]. This may be significant given the importance of neutrophils in the host response to systemic fungal infection.

Analysis of pharmacokinetic parameters in RTD-1 treated mice revealed that the maximum plasma concentrations achieved in the multiple i.p. dosing scheme employed were less than 1% of that required to kill either organism in the presence of 50% mouse serum [60]. These data demonstrate that the effect of RTD-1 in resolving systemic candidiasis is not the result of direct fungal killing, but rather peptide-mediated augmentation of the host antifungal response. While the mechanistic details underlying the observed efficacy in vivo have yet to be revealed, a common feature of RTD-1 in multiple models is the modulation of dysregulated inflammation. This effect has been obtained in RTD-1 treatment of endotoxin-mediated lung injury [123], *P. aeruginosa* lung infection [119,124], and pulmonary inflammation in a murine model of SARS coronavirus infection [65]. These data suggest that cyclic θ-defensins represent a novel approach for developing HDPs as host directed agents that enhance the host response to fungal infection.

### 3.4. Immunomodulatory Peptides

The concept of host directed immunomodulation for treatment of infection has gained attention in recent years. Two examples are immunotherapeutic peptides, Raltecimod™ and Zadaxin™, both of which regulate systemic inflammation in the host. Raltecimod^TM^ (AB103) developed by Atox Bio, is a CD28 mimetic that antagonizes CD28 cell signaling [66,67]. It is efficacious in mouse models of toxic shock, sepsis, and Gram negative bacterial peritonitis by promoting bacterial clearance and preventing septic shock. Zadaxin^TM^ is a synthetic version of the 28 amino acid human peptide thymosin α 1. The peptide regulates multiple immune cell subsets by stimulating the production of cytokines and has been approved in 35 countries for treatment of hepatitis B and C and also shows promise for treatment of severe sepsis [68,69,70].

### 3.5. Antifungal Peptide Mimics

A promising approach, circumventing limitations of HDPs per se, has been the design and evaluation of HDP mimetics [71,125,126]. These nonpeptidic molecules, based on a meta-phenylene ethynylene core [127], a peptide mimic inspired by membrane binding properties of the frog skin peptide magainin II [128]. One analog, compound **C4** (Table 3) was shown to permeabilize *C. albicans* yeast and hyphal cell membranes, inducing ATP release and propidium iodide uptake [71]. Further, **C4** was fungicidal in vitro and in vivo and was highly efficacious in promoting long term survival of immunosuppressed mice challenged with *C. albicans* SC5314. Importantly, initial toxicity studies showed that **C4** and related compounds have low in vitro and in vivo toxicities, demonstrating selectivity of a new class of antifungals [126].

## 4. Conclusions and Future Perspectives

Despite warnings of emerging MDR fungal pathogens, such as *C. auris*, only recently have there been significant national and international efforts devoted to antimicrobial resistance, only a fraction of which has been focused on systemic mycoses. Nevertheless, pockets of the scientific community are employing novel strategies to develop the next class of antifungal agents and novel therapeutic approaches. In this regard, peptide mimetics certainly hold promise. Further, there is growing evidence that HDP-inspired peptides that augment the host antifungal response and modulate dysregulated inflammation will contribute to the list of possible therapeutic solutions urgently required. Given the remarkable increase of peptides entering clinical trials in recent years (https://webs.iiitd.edu.in/raghava/thpdb/index.html), there is reason to be optimistic that peptides and peptide mimics will contribute to a much needed expansion of the clinical anti-infective armamentarium.

## Figures and Tables

**Table 1:** Annual worldwide fungal infections.

Fungal Disease	Estimated Cases	Mortality Rate	References
Candidiasis	400,000/year	10–75%	[1,2,4]
Aspergillosis	200,000/year	30–95%	[1,4]
Cryptococcosis	1,000,000/year	20–70%	[1,4]

**Table 2:** Approved antifungals for clinical usage.

Antifungal Class	Fungal Targets	Mechanism of Action	Limitation	References
Azoles	*Aspergillus, Candida, Cryptococcus*	Interference with ergosterol biosynthesis via 14α-demethylase inhibition	Fungistatic	[4,5]
Polyenes	*Aspergillus, Candida, Cryptococcus, Histoplasma, Blastomyces*	Ergosterol extraction from plasma membrane	Toxicity	[4]
Echinocandins	*Aspergillus, Candida*	β-1,3-glucan synthase inhibition	Poor oral bioavailability	[7]

**Table 3:** Representative host defense peptides (HDPs) and mimics.

Compound	Sequence/Structure	Structural Motif	Activity	Reference/Database
Hst5 (histatin)	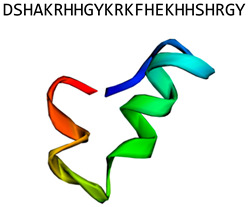	α-helix	Internalized after binding to fungal cell wall. Induces oxidative stress, affects ionic balance and mitochondrial function	Chemspider: 17289075 [33]
LL-37 (human cathelicidin)	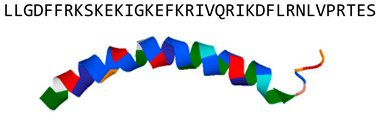	α-helix	Disrupts fungal membrane and modulates host responses in vitro	PDB: 2K6O [20,37,38,39,40,41,42,43,44,45]
Protegrin (porcine cathelicidin)	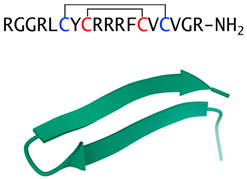	Cysteine-rich β-sheet, two disulfide bridges	Fungicidal in vitro, cytotoxic and hemolytic to mammalian cells	PDB: 1PG1 [46]
Indolicidin (bovine cathelicidin)	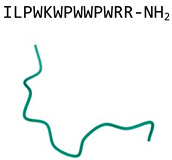	Extended chain containing five tryptophans	Antifungal activity, toxic in mammals	PDB:1G89 [47]
HNP-1 (α-defensin)	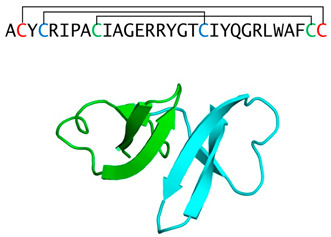	Tri-disulfide stabilized β-sheet dominated monomers that from non-covalent dimers	Fungicidal in vitro, modulates host responses in vivo	PDBE: 3hj2 [48,49,50,51]
HBD-2 (β-defensin)	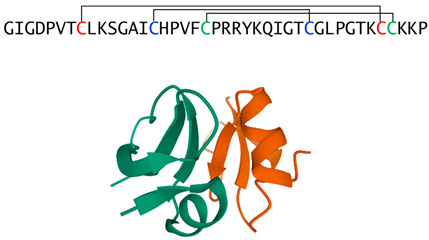	Tri-disulfide stabilized monomers that from non-covalent dimers	Fungicidal in vitro, modulates host responses in vivo	PDB:1FD4 [52,53,54,55]
IDR-1018 (bactenecin derivative)	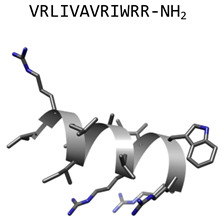	α-helix	Modulates cytokines in bone marrow-derived macrophages stimulated with heat-killed *C. albicans*	[56,57]
hLF1-11 (human lactoferrin cryptide)	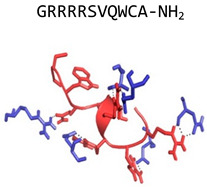	First 11 N-terminal amino acids of the human lactoferrin loop peptide	Fungicidal in vitro, inhibits *C. albicans* filamentation and reduces TNFα and IL-6 in vivo	[58,59]
RTD-1 (θ-defensin)	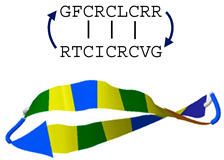	Tri-disulfide β-stranded macrocycle	Permeabilizes fungal cells and induces oxidative stress in vitro, enhances fungal clearance in vivo	PDB: 2LYF [9,60,61,62,63,64,65]
AB103; Raltecimod (CD-28 mimetic)	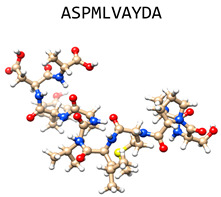	D-Ala1, D-Ala10 CD-28 mimetic	Modulates host responses in vivo	Chemspider: 58828035 [66,67]
Zadaxin	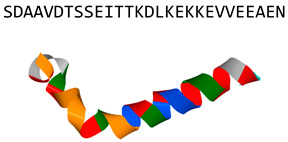	α-helix; synthetic version of human peptide thymosin α 1	Modulates host responses in vivo	PDB: 2L9I [68,69,70]
C4; Compound 4 (mPE-derived)	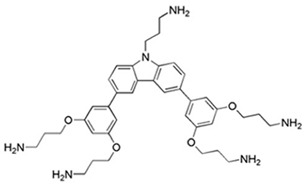	Peptidomimetic derived from the magainin-inspired mPE	Disrupt fungal cell membrane in vitro, fungicidal in oral and systemic candidiasis in vivo	[71,72]
